# Fifty years of child height and weight in Japan and South Korea: Contrasting secular trend patterns analyzed by SITAR

**DOI:** 10.1002/ajhb.23054

**Published:** 2017-08-23

**Authors:** T. J. Cole, H. Mori

**Affiliations:** ^1^ Population, Policy and Practice Programme UCL Great Ormond Street Institute of Child Health London United Kingdom; ^2^ Senshu University Tokyo Japan

## Abstract

**Objectives** Japanese and South Koreans have traditionally been shorter than Europeans, but have recently become appreciably taller. The aim was to quantify the secular trend patterns in height and weight growth in the two countries over 50 years using the SITAR growth curve model.

**Methods** Data on mean height and weight by sex in 1‐year age groups from 1 to 20 years were obtained by decade in South Korea (1965–2005) and Japan (1950–2010). The data were analyzed using SITAR (SuperImposition by Translation And Rotation), which estimates a mean curve and three adjustments–size, timing and intensity–reflecting how the individual surveys differ from the mean. A sensitivity analysis compared results for the Japanese data based on cohort as well as period.

**Results** Growth patterns in the two countries changed dramatically over the study period, affecting not only height and weight but also developmental age, in that the growth period advanced in timing and shrank in duration. SITAR fitted the data well. The trends were larger in South Korea than Japan, and puberty timing in Japan stabilized by 1970. Most of the height increment seen in adults had already accrued by age 1.5 years, whereas the adult weight increment accrued throughout childhood.

**Conclusions** The secular height trend in these countries represents increased growth in the long bones during infancy, so it can be viewed as the inverse of stunting. There are striking country differences in growth pattern, but they are not easily explained by differences in national income, diet or lifestyle.

## 1 INTRODUCTION

The Japanese have traditionally been much shorter in height than their European counterparts, particularly Scandinavians, though they have experienced a dramatic secular increase in height since World War II (Matsumoto, 1982). Tanner, Hayashi, Preece, and Cameron (1982) showed this to be due primarily to an increase in leg length, possibly arising from the shift from rice to a dairy‐based diet during the 1970s.

South Korea is another East Asian country whose inhabitants are materially shorter than in the West—indeed in 1978 South Korean children were shorter even than the Japanese (Y. S. Kim, 1982). However they, like the Japanese, have experienced a steep increase in height in recent decades, and they are now taller than the Japanese (J. Y. Kim et al., 2008; Mori, 2017). Furthermore, South Koreans are among the tallest peoples in the Asian continent (Schwekendiek & Jun, 2010), and South Korean women are 20 cm taller now than they were a century ago, a larger increase over the period than any other group (NCD Risk Factor Collaboration, 2016).

It is of interest that these two neighboring countries have, over a similar period of time, experienced remarkable secular increases in height, and yet in detail the patterns of change have been very different, with the South Koreans “leap‐frogging” over the Japanese, by starting out shorter and ending up taller.

The secular trend in height as seen in children combines two distinct processes: not only does height itself increase, but the age at peak height velocity in puberty gets earlier, due to accelerated maturation. Thus, over a period of time, the curve of mean height plotted against age shifts upwards, and at the same time it shifts to the left.

The recently developed SITAR growth curve model provides a way to examine these components of the secular trend in more detail (Cole, Donaldson, & Ben‐Shlomo, 2010). It does this by explicitly modeling the two separate components of the growth curve: increasing size and accelerating maturation. It can also be applied to “growth curves” based on mean height by age in representative cross‐sectional samples, where “individuals” correspond to particular surveys.

The way SITAR works is to summarize the pattern of growth in individuals as a mean growth curve plus a set of adjustments for individuals that transform the mean curve to match their individual curve. The three transforming adjustments reflect differences between individuals in size (mean height), timing (or tempo, similar to age at peak height velocity), and intensity (similar to peak height velocity).

When modeling the secular trend, the adjustment of size represents the change in adult height, while the adjustments of timing and intensity reflect the change in rate of maturation. Thus, the secular trend can be decomposed into three separate components. The technique represents an extension of the approach by Ali, Lestrel, and Ohtsuka (2000) who analyzed growth curves of Japanese height over eight decades using kernel regression, and identified turning points on the curves reflecting age at take‐off and age at peak velocity.

This article aims to decompose the secular trends in height and weight among Japanese and South Korean children and young people over long periods of time (40 years in South Korea, 1965–2005, and 60 years in Japan, 1950–2010), and to show how the trends can be explained in terms of country‐specific changes in SITAR size, timing, and intensity.

## 2 METHODS

### 2.1 Data

Mean values of height (cm) and weight (kg) for boys and girls, tabulated in 1‐year age groups from 1 to 20 completed years, were obtained from national samples of child anthropometry. Half a year was added to each year group age to reflect mean age. The data represent *periods*, i.e., children measured in particular years, not *cohorts* of children born in the same year. South Korean data were available only for the years 1965, 1975, 1984, 1997, and 2005 (J. Y. Kim et al., 2008), while the Japanese data came from annual National Nutrition Surveys carried out between 1949 and 2011 by the Japanese Ministry of Health and Welfare (latterly Ministry of Health, Labor and Welfare)—see Funatogawa, Funatogawa, Nakao, Karita, and Yano (2009) for details. The South Korean surveys were very large, with each age group mean based on 15,000 to 60,000 children, while the Japanese samples were much smaller, ranging in size from 500 to 3500 for all ages 1–20 years. To make the two datasets more similar, the Japanese data were merged in 3‐year groups at 10–year intervals as follows: 1949–1951, 1959–1961, 1969–1971, 1979–1981, 1989–1991, 1999–2001 and 2009–2011.

**Figure 1:**
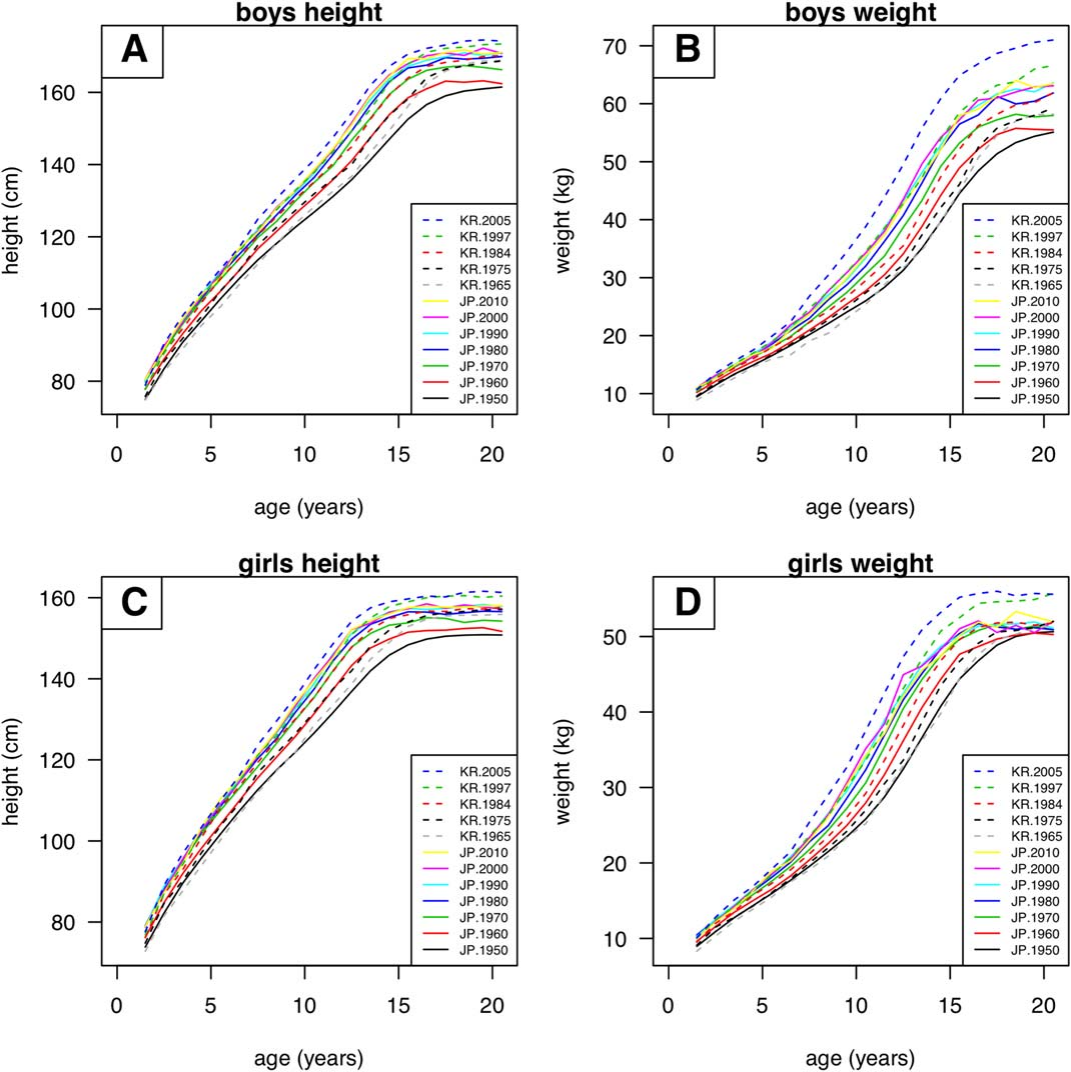
Growth curves for height (left) and weight (right) by country and year of measurement, boys (above) and girls (below)

Supporting Information Tables S1–S4 give the mean values by age group, sex, country and year of measurement, and Figure 1 shows the corresponding period growth curves for height and weight (discussed in more detail later).

### 2.2 SITAR growth curve model

SITAR (SuperImposition by Translation And Rotation) is a shape‐invariant mixed effects growth model (Cole et al., 2010). It fits a mean curve to the data as a natural cubic B‐spline, on the assumption that the growth curves of individuals (i.e., individual surveys here) differ from this mean curve shape in just three ways, corresponding to three subject‐specific transformations. The three transformations are size, i.e., how tall individuals are relative to the mean; timing, i.e., their relative timing of the pubertal growth spurt based on the age at peak velocity, and intensity, the relative rate at which they pass through childhood and puberty. Intensity is effectively a measure of accelerated developmental age, in that biological time passes faster when development is advanced and slower when it is delayed.

The geometric impact of the three SITAR transformations on individual growth curves, reflecting how they differ from the mean curve, is as follows: they are shifted up/down (size, reflecting the curve being above or below average size) or left/right (timing, corresponding to early or late timing), and the age scale is stretched/shrunk (intensity, this transformation having the effect of making the curve respectively shallower or steeper, a positive value indicating advanced growth). The transformations are in units of cm or kg (for size), years (timing) and percentage (intensity). So for example a height curve with size +5 cm, timing −0.5 years and intensity +4% indicates that the individual is 5 cm taller as an adult, peaks in velocity 6 months earlier and completes growth 4% faster than the mean curve. Using their fitted transformations, individual curves can be back‐transformed to match the mean curve; if the model fits well the adjusted curves will all be superimposed (hence the name SITAR).

The SITAR model was fitted in the statistical language *R* version 3.3.2 (R Core Team, 2015) using the *sitar* package version 1.0.9 (Cole, 2016). Four separate models were fitted, for height and weight in boys and girls, with the seven surveys by decade for Japan and five for South Korea treated as 12 individual growth curves. The sexes were analyzed separately due to their differing mean curve shapes.

The transformations size, timing and intensity were estimated as random effects, with zero mean, included in the model along with corresponding fixed effects. For presentation intensity was multiplied by 100 to convert it to a percentage. The goodness of fit was measured as the percentage of variance explained by adding the random effects to a simpler model fitting just the mean curve (Cole, Pan, & Butler, 2014).

### 2.3 Optimizing the SITAR model

The SITAR models were optimized by choosing the degrees of freedom for the cubic B‐spline curve to minimize the Bayesian Information Criterion or BIC (Schwarz, 1978). The fitted curves had either 6, 7, or 8 degrees of freedom, with knots placed at the corresponding age quantiles.

Other models were explored, optimized by transforming the age and/or size scales. The main model summarizes the differences between surveys on the age and size scales in absolute terms, e.g., shifting the height growth curve upwards by say 5 cm, or shifting it left by say 0.5 years; these adjustments are the same at all ages. If instead age and/or size are logarithmically transformed, then the predicted effects at each age are proportional rather than absolute, e.g., shifting the curve up or shifting it left by say 4%; in absolute terms these adjustments are smaller at younger ages. The square root transformation is intermediate between linear and log, and hence is midway between absolute and proportional effects. All three transformations were explored in sensitivity analyses, with the fitted curves back‐transformed for presentation purposes.

### 2.4 Velocity curves

The first derivative of the (back‐transformed if necessary) growth curve shows the difference in size by year of age, a measure of growth, which for convenience is here termed the “velocity” curve (although based on cross‐sectional rather than longitudinal data). The area under the velocity curve corresponds to the total increment in size, and so the curve is useful to illustrate how the SITAR effects of timing and intensity impact on size at each age. Sensitivity analyses explored which transformations of the measurement and age scales best captured the secular changes in the velocity curve.

### 2.5 Cohort versus period

Height and weight in the two countries were tabulated by age and year of measurement. Analysing the data in this form assumes that the growth pattern depends only on the *period*, the time when the measurement was made. However, the time when the children were born—their *birth cohort*—could also be relevant, so that both year of birth and year of measurement may affect the growth curve's shape.

The Japanese data being available annually allowed growth curves to be constructed both by cohort (i.e., successive years of age for a given year of birth cohort correspond to successive years of measurement)—see Funatogawa, Funatogawa, Nakao, Karita, and Yano (2009)—as well as by period. (Note though that this was not possible for the Korean data, which were available only by decade of measurement, so the primary country comparison here is necessarily based on period.)

If one assumes that the secular trend in growth is monotonic (i.e., that it does not change direction at any point), then the cohort and period approaches will lead to broadly similar results—children of later‐born cohorts and later measurement years will be taller and heavier than for earlier cohorts and measurement years. However, it is not clear how the trends in the corresponding SITAR parameters might differ when viewed the two ways.

As a sensitivity analysis the Japanese growth curves were analyzed both by period and by cohort, to see how the SITAR growth parameters were affected; to do this the “period” curve for a given measurement date was compared with the “cohort” curve for the birth cohort born 11 years earlier (e.g., those born in 1939 were compared with those measured in 1950; this ensured that in mid‐1950 the mean age of the cohort was 11.0, matching that for the period dataset). The cohort data were inevitably incomplete for the early and late years.

## 3 RESULTS

### 3.1 Height and weight in Japan and South Korea

Figure 1 shows the growth curves for height (left) and weight (right) in boys (above) and girls (below). Each quadrant contains 12 curves, i.e., the seven surveys in Japan (solid lines) and five in South Korea (dashed lines). There was a clear secular trend to increasing height and weight over time, with Japan shortest and lightest in 1950, and South Korea tallest and heaviest in 2005.

There was also an obvious secular trend to earlier maturation, with the curves shifting to the left over time. To express these differences in terms of size, timing and intensity, separate SITAR models were fitted to each quadrant of Figure 1.

**Figure 2:**
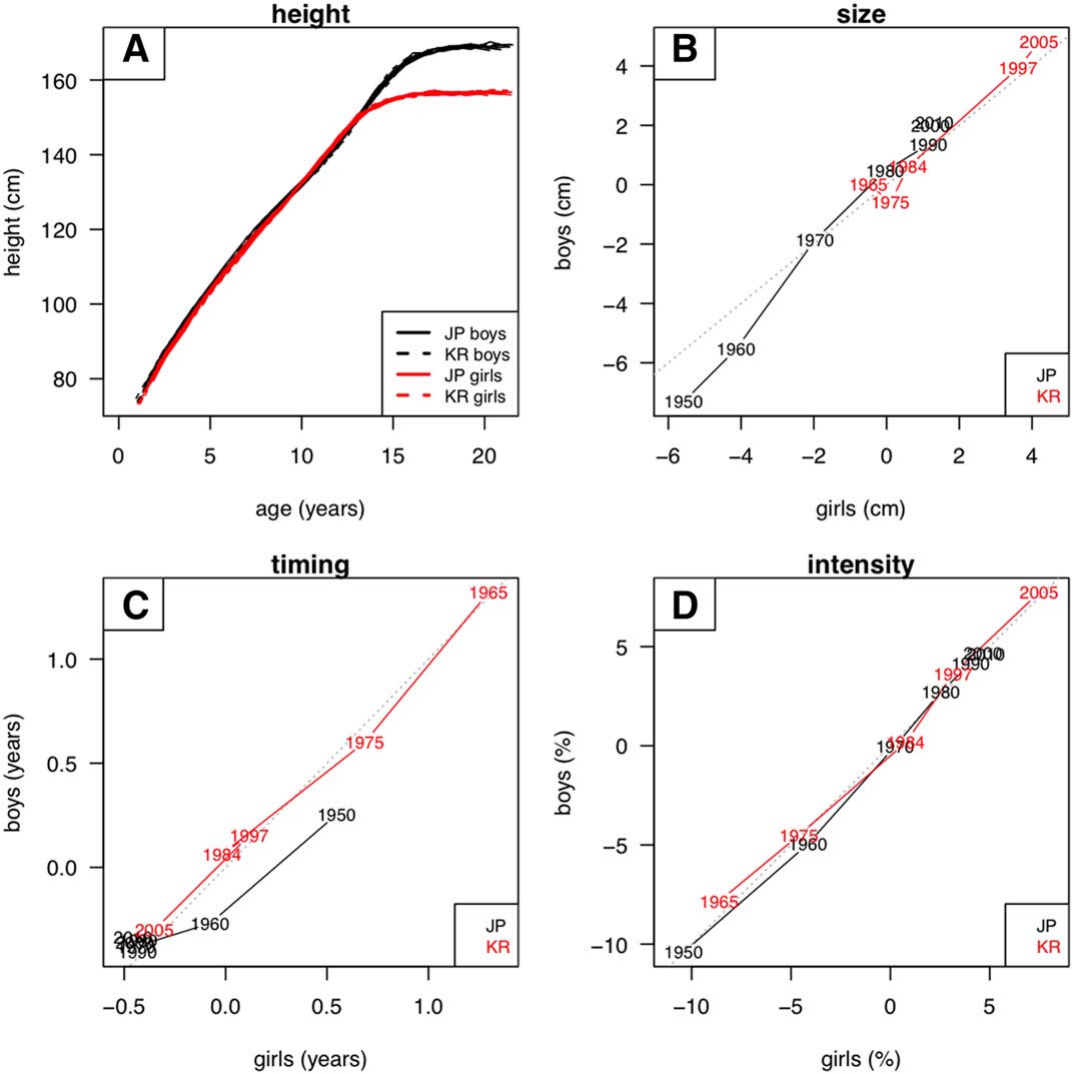
SITAR summary of height growth by sex, country and year of measurement: (A) SITAR‐adjusted height curves (boys black, girls red); (B) changes in size (cm) for boys plotted against girls (Japan black, Korea red); (C) changes in timing (years) similarly; and (D) changes in intensity (%) similarly. See text for details

Figure 2a illustrates the model fit for height in boys and girls, where the 12 boys' curves are shown in black and the 12 girls' curves in red, and the two countries are indexed by line type. The individual curves by sex are all superimposed, with 97% of the variance in Figure 1a,c explained, indicating that the models fit well. The mean curve shapes for the two sexes, with the girls (red) spurting earlier but ending up shorter than the boys (black), are clearly visible.

The other three quadrants in Figure 2 illustrate the random effect estimates of SITAR size, timing and intensity for each of the 12 surveys, indicated by the year label and country color (Japan black, South Korea red), and joined by lines to indicate the temporal trend. The values for boys and girls are plotted against each other, and the diagonal line of equality demonstrates that the values for the two sexes are very similar.

Mean height size (Figure 2b) increased by about 8 cm in Japan and 5 cm in South Korea (for both sexes) over the period of study. Note that this is after adjusting for concurrent trends in timing and intensity, so it reflects the increase in adult height. However, the bulk of the increase occurred before 1980 in Japan and after 1984 in South Korea (in 1980/1984 the two countries were similar in height). In addition Japan showed no change from 1990 to 2010, an indication that the secular trend had stopped, whereas in South Korea mean height increased slightly from 1997 to 2005.

Figure 2c compares the timing of puberty in the different surveys, where again the two sexes were in close agreement. However, there was a dramatic difference between the two countries in that Japan showed virtually no change in timing after 1970, whereas in South Korea puberty occurred 6 months earlier in 2005 than in 1997. Ali et al. (2000) also found that in Japan, age at peak height velocity plateaued after 1970.

Finally Figure 2d compares growth intensity in the two countries, where the sexes are again almost identical. Intensity increased by 15% over the period, this indicating by how much the duration of growth had shrunk. However, the increase was more rapid in South Korea, occurring over a 40 year period as against 60 years in Japan.

**Figure 3:**
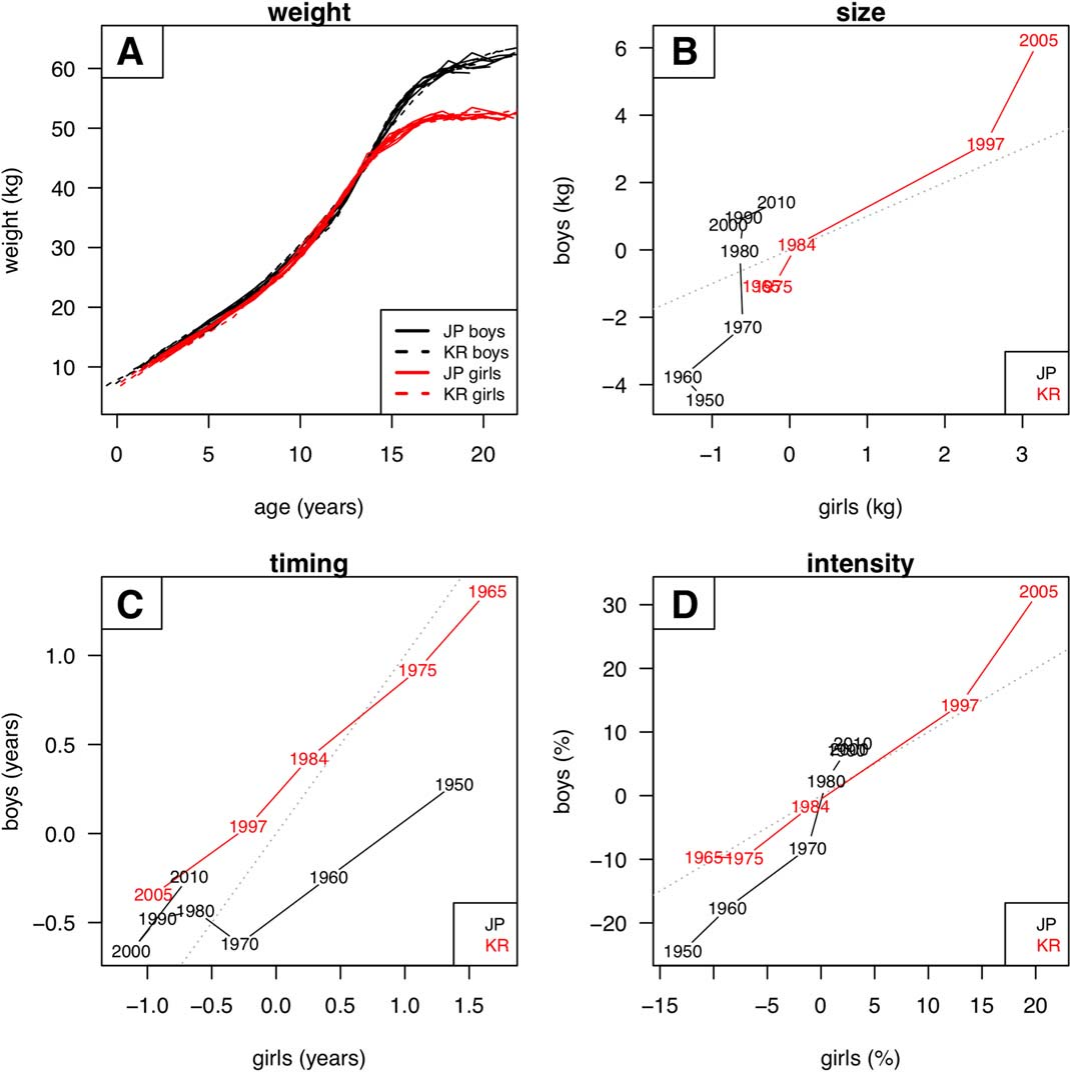
SITAR summary of weight growth by sex, country and year of measurement: (A) SITAR‐adjusted weight curves (boys black, girls red); (B) changes in size (kg) for boys plotted against girls (Japan black, Korea red); (C) changes in timing (years) similarly; and (D) changes in intensity (%) similarly. See text for details

The results for weight are shown in Figure 3. Again the SITAR models fitted well (Figure 3a), with 97% of variance explained. There was more sexual dimorphism than for height, with the size, timing and intensity effects in Figures 3b‐d less close to the line of equality. Boys increased in size over the period by twice as much as girls (6 vs. 1.5 kg in Japan, 7 vs. 4 kg in South Korea). For timing (Figure 3c) the results again showed a greater effect in South Korea than Japan, an advance of 2.5 years, and within Japan the effect was greater in girls than boys. Weight intensity (Figure 3d) showed a similar pattern to size, increasing more in South Korea than in Japan, and in boys more than girls.

### 3.2 Height and weight increment by age

**Figure 4:**
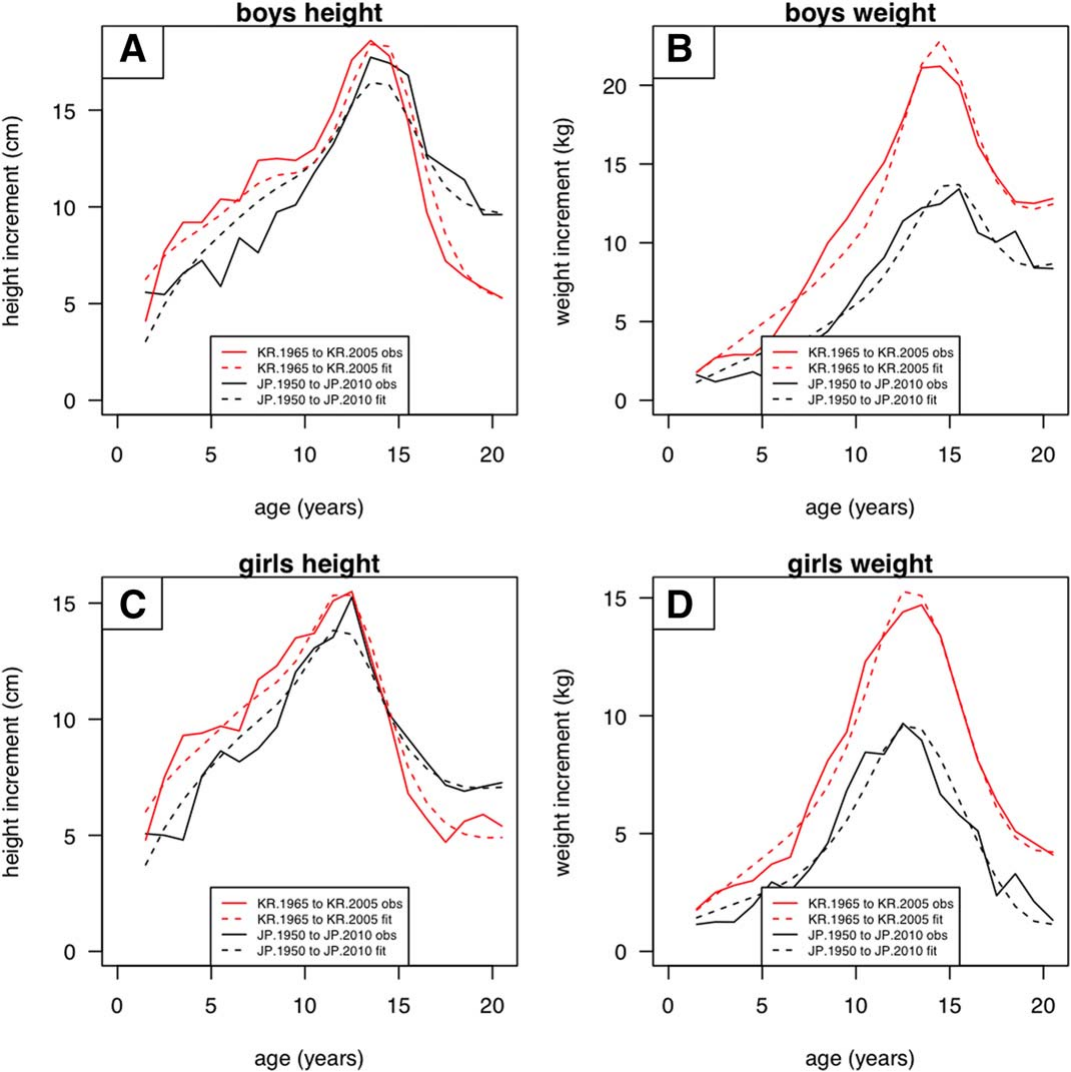
Increments by age for height (left) and weight (right) in Japan (1950 to 2010, black) and South Korea (1965 to 2005, red), boys (above) and girls (below). The increments are shown observed (solid lines) and predicted by SITAR (dashed lines)

Figure 4 explores the increments in height and weight by age and sex in the two countries over the period of study, i.e., 60 years for Japan and 40 years for South Korea. The increments are strongly age‐dependent, depending as they do on the underlying changes in size, timing and intensity; thus, the maximum increments occurred in puberty, at around 14 years in boys and 12 years in girls. Figure 4 shows both the observed increments and those predicted by the SITAR models, and the two agree well. The increments at age 20 indicate the adult secular trends, and the adult height increments were appreciably greater for Japan than South Korea, reflecting how short the Japanese were in 1950. Conversely the weight increments were greater in South Korea.

There is a particular interest in the size of the height increment in early life, as it has been suggested that the size increment as seen in adults is accrued as early as age 2 (Cole, 2000a). The youngest infants (mean age 1.5 years) were 5 cm taller at the end of the period than at the start (Figure 4a,c). This is the same as the adult increments in South Korea, but slightly less than in Japan (10 cm in boys and 7 cm in girls). So in South Korea the 5 cm increment in adult height had already been accrued by 1.5 years, whereas in Japan an extra 2–5 cm in height was gained during childhood after infancy.

With weight, the adult increments were much smaller for girls than boys in both countries. The early life increments were all considerably smaller for weight than for height. For Japanese girls the adult weight increment matched that in early life, while for South Korean girls, and boys in both countries, it was greater.

### 3.3 Secular trends in height velocity

**Figure 5:**
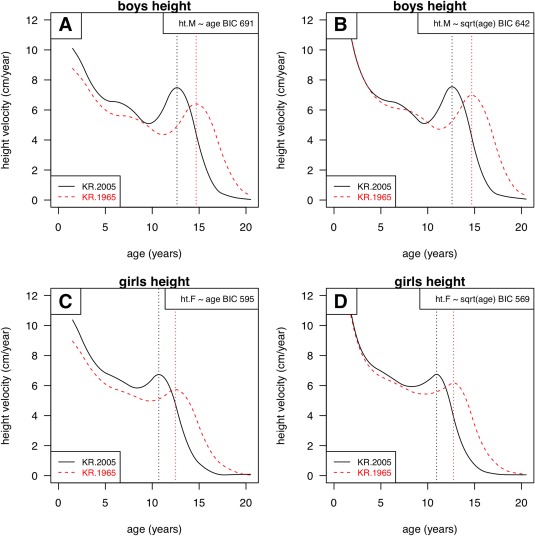
Changes in height velocity curve shape for Korean boys (above) and girls (below), from 1965 (red dashed lines) to 2005 (black solid lines). The curves are predictions from SITAR models of height on age (left) and height on √age (right), where the latter models have lower BIC and fit better

**Figure 6:**
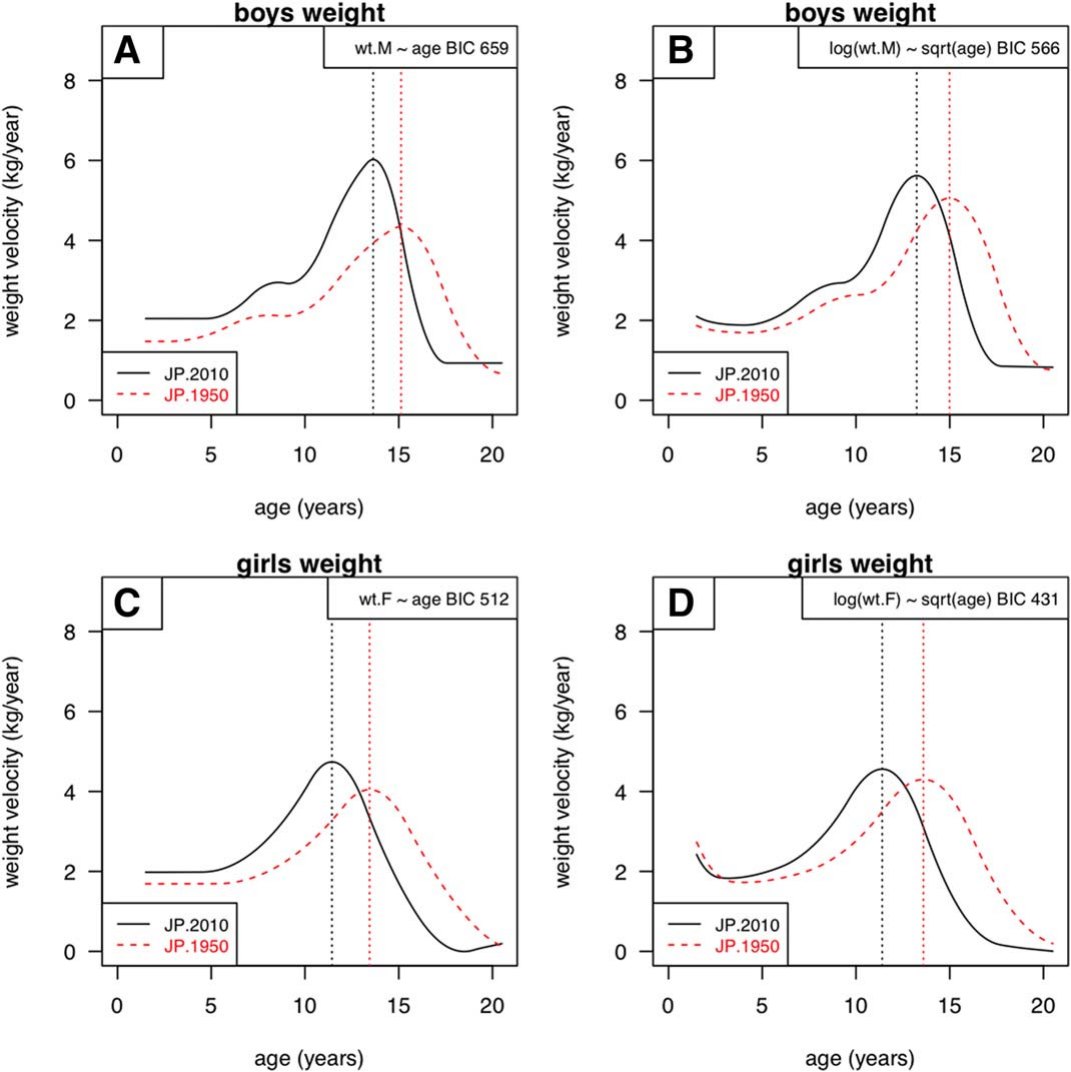
Changes in weight velocity curve shape for Japanese boys (above) and girls (below), from 1950 (red dashed lines) to 2010 (black solid lines). The curves are predictions from SITAR models of weight on age (left) and log weight on √age (right), where the latter models have lower BIC and fit better

Figures 2, 3, 4 confirm the good fit of the model to changes in size over time. A more subtle aspect of model fit is the shape of the predicted velocity curve and how it changes over time. This is explored in Figure 5 for height velocity in South Korea, and in Figure 6 for weight velocity in Japan.

Figure 5 contrasts two alternative SITAR height models (left and right) for boys (above) and girls (below). The models on the left are those for height versus age as already discussed, while on the right are better fitting models with age on the square root scale, where the BIC is 30–50 units smaller (and hence better fitting). The figures contrast the predicted height velocity curves in 1965 and 2005, showing how puberty advanced over time.

The simpler models (left) imply that before age 10 velocity was greater in 2005, whereas the optimal models (right) show it was unchanged from 1965. This reinforces the impression from Figure 4 that height velocity after infancy did not change over the period. The corresponding figure for Japan (not shown) indicated that height velocity up to age 10 was slightly greater in 2010 than in 1950, which accords with the observed secular increase in height in Japan beyond 1.5 years.

Figure 6 looks at weight velocity in Japan similarly. It is clear in all four quadrants that weight velocity before age 10 was considerably greater in 2005 than in 1965, thus explaining the observed secular trend in weight. However, the better fitting models (log weight and square root age, BIC ∼80 units less, on the right) show that this applied much less to peak weight velocity in puberty, which did not change materially.

### 3.4 Cohort versus period

**Figure 7:**
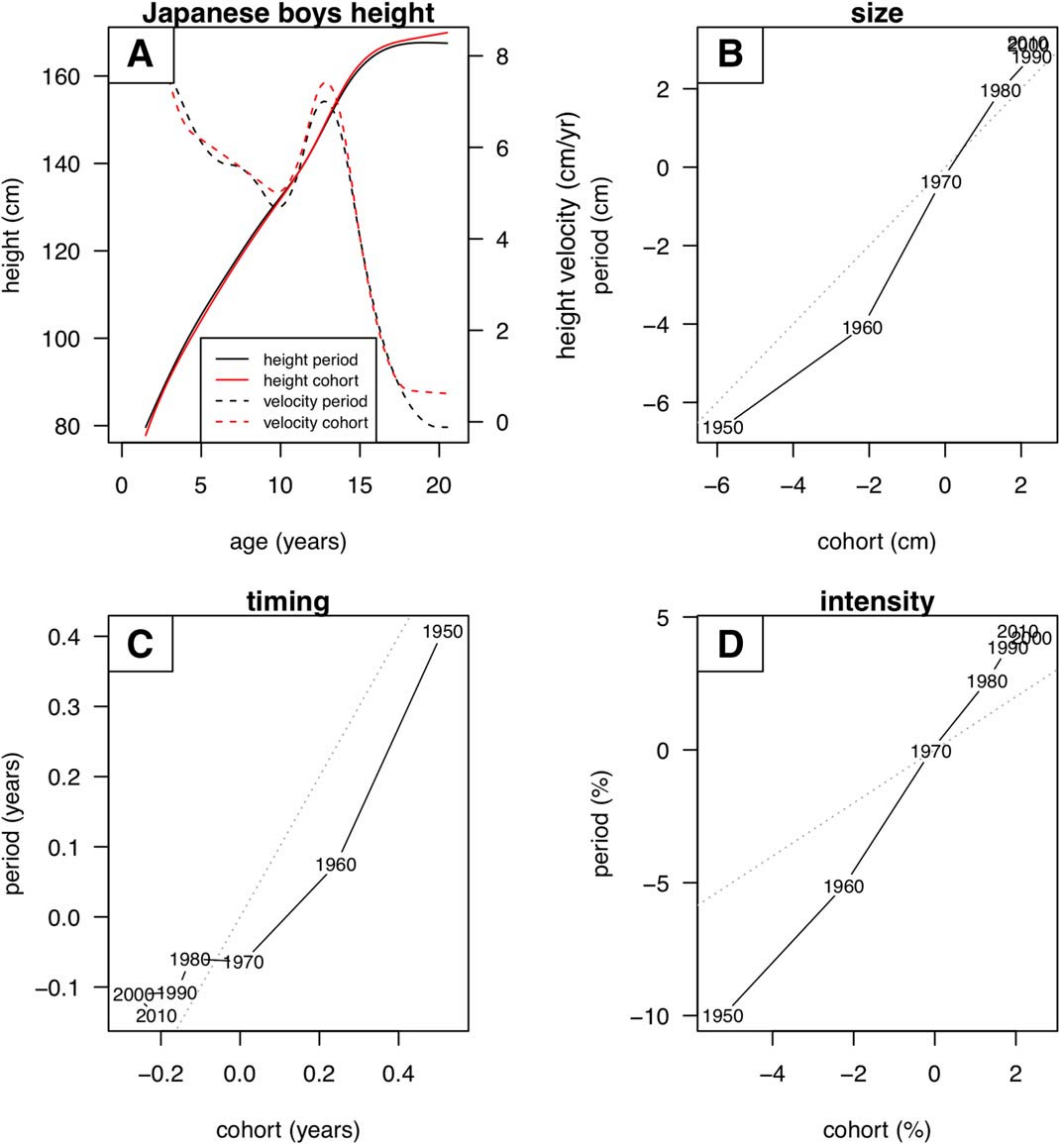
SITAR summary of Japanese boys height by year of measurement, with growth curves constructed by period and by cohort: (A) mean curves (period black, cohort red; height solid, velocity dashed); (B) changes in size (cm) for period plotted against cohort; (C) changes in timing (years) similarly; and (D) changes in intensity (%) similarly. See text for details

As a sensitivity analysis, Figure 7 compares the Japanese boys height growth curves as defined by cohort and by period.

Figure 7a contrasts the two mean curves, where the cohort curve (solid red line) is lower than the period curve (solid black line) at younger ages but higher at older ages. This is because within each cohort, the younger and older ages correspond to earlier and later measurement dates, whereas the corresponding period data are all measured at the same time. The net effect is to make the mean cohort curve steeper, so the peak height velocity for cohort in Figure 7a (dashed red line) is greater than for period (dashed black line). This is equivalent to increased intensity for the cohort curve, i.e., the age scale is foreshortened.

Figures 7b‐d compare the corresponding trends over time in the SITAR effects for size, timing and intensity by cohort and period. Although there are differences, the size and timing effects are broadly similar the two ways, lying close to the dotted line of equality. However, the intensity trend is much steeper for period, the range of values being twice that for cohort (15% vs. 7%). A similar though less extreme pattern was seen for girls height and boys and girls weight (data not shown). This confirms that the main difference between cohort and period curves lies in the SITAR intensity parameter, the secular trend in intensity for the cohort curves being as little as half that for the period curves.

## 4 DISCUSSION

The results demonstrate the dramatic secular trends in child height and weight that have taken place in Japan and South Korea during the past 40–60 years. The trend has been appreciably greater in South Korea, where the children were shorter and lighter than in Japan in 1965, but taller and heavier in 2005.

The results also show how well SITAR summarizes the secular changes in growth pattern, with over 97% of the variance between surveys being explained by differences in mean size, growth timing and intensity. This emphasizes that the secular changes over time that occurred in height and weight, which one might think were simply a reflection of increasing body size, were also driven by similar changes on the developmental age scale—earlier puberty and faster passage.

The results demonstrate in three distinct ways how well SITAR represents the changing growth patterns in the two countries. In Figures 2a and 3a, the individual survey growth curves are all superimposed after SITAR adjustment, with the curves for the two sexes clearly distinct. In Figure 4 the increments over 40 or 60 years are well characterized by the SITAR models, and in Figures 5 and 6 the changes in velocity curve shape over time are convincingly captured.

The SITAR analysis adjusts mean height and weight for differences in the timing and intensity of growth. For this reason the mean size results in Figures 2b and 3b directly reflect the corresponding secular trends in adult size, i.e., after growth has stopped. For height, they show that in Japan the growth pattern did not change after 1997, whereas in South Korea it continued changing up until 2005.

A similar picture emerges for weight, with much larger secular changes in growth pattern among South Koreans than Japanese. However weight, unlike height, is still increasing in Japan. There is also sexual dimorphism in the weight growth patterns, with girls changing less than boys in terms both of size and intensity. This confirms the observation by Maruyama and Nakamura (2015) that the Japanese gender difference in BMI secular trend was due to a difference in the weight trend not the height trend.

Tanner et al. (1982) pointed out that the Japanese secular height trend from 1957 to 1977 was driven almost entirely by an increase in leg length. Ali et al. (2000) studied secular changes in Japanese leg length for birth cohorts from 1943 to 1978, showing a steep rise in leg length as a percentage of height from 1943 to 1968 but a plateau thereafter. In addition they showed that this percentage increased with age until puberty, i.e., 13 years in boys and 11 years in girls, and then either stabilized (girls) or fell slightly (boys). Thus, in terms of length (cm) the trend in leg length was greater than the trend in height (and even greater than the trend in sitting height) up to puberty (though not thereafter). So the height trend was until puberty primarily a leg length trend.

The secular increment in adult height (i.e., at age 20) amounted to 8–10 cm in Japan and 5 cm in South Korea (Figure 2b). Figure 4 shows how this increment developed through childhood, and the pattern is striking; 5 cm of it, i.e., the majority, was already apparent at age 1.5 years, the mean age for the youngest age group. The increment appeared to be greater at later ages, but this reflected only more advanced maturation. In South Korea the peak increment was at age 12–14 and exceeded 15 cm, but by adulthood it had shrunk to 5 cm, the same as at age 1.5. So there was no net increase in height after infancy. By contrast in Japan the adult increment of 8–10 cm was greater than the 5 cm increment at age 1.5, showing some growth in childhood after infancy, so the two countries differed in this respect. The secular increment in length at birth is known to be very small; in Shanghai for example mean birth length increased from 1985 to 2005 by just 0.2 cm (Zhang & Li, 2015). So, it is reasonable to state that in South Korea the secular increase in adult height occurred during the first 18 months of postnatal life, driven primarily by extra growth in the long bones of the leg, which confirms and refines the previously suggested 2 years (Cole, 2000a).

This, it should be noted, is the exact opposite of the malnutrition state of *stunting*, where infants in the first 1000 days of prenatal and postnatal life fall behind in terms of linear growth (Dewey, 2016). It is tantalizing to think that the beneficial changes over time that have driven the secular trend to increased stature may well be the inverse of the factors that are driving linear growth retardation in millions of poor infants.

The secular increase in adult weight was also substantial, but rather greater in boys than girls and in South Korea more than Japan (Figures 3b and 4). In addition the increase accrued throughout childhood and puberty rather than being restricted to infancy (Figure 6b,d). It is for this reason that the best data transformation for size (as seen in Figures 5 and 6) was linear for height and log for weight; the absolute increment in height was the same in early and adult life (Figure 4), whereas for weight the proportional increment was closer to constant—this corresponds to the log transformation (Cole, 2000b).

The best data transformation for age was the square root (Figures 5 and 6). In principle one would expect differences in developmental age to be multiplicative, corresponding to the age scale being stretched or shrunk, and this in turn corresponds to the log age scale, as seen in the original SITAR publication (Cole et al., 2010). Conversely the linear age scale would imply the whole growth curve being shifted left or right, which could fit poorly in infancy with age becoming negative. The square root scale is a compromise between linear and log, implying that differences on the developmental age scale are somewhere between multiplicative and additive.

The sensitivity analysis showed that secular trends in the SITAR size and timing parameters were broadly the same whether based on cohort or period curves. However, the cohort‐based intensity parameter changed over time only half as fast as the period‐based intensity. This can be explained by reference to Figure 7a, where the mean cohort curve (solid red line) is slightly steeper than the mean period curve (black). This is because within each cohort, the younger and older ages correspond to earlier and later measurement dates, whereas the corresponding period data are all measured at the same time. Expressed the other way round, the period curves include some cohort information as their data are spread across 20 birth years, with the younger children born later and the older earlier. The net effect is that the secular trend in intensity based on the period data is up to twice that for the cohort data, and hence provides a biased estimate of the cohort‐based trend.

However, it is important to emphasize that the SITAR analysis, in summarizing the secular trend, does not claim to provide any extra insight as to whether the trend is driven by cohort or period differences. Probably both apply, in that children's individual growth patterns are inherited in early life (i.e., a cohort effect) but are subsequently influenced by the changing environment (a period effect). The two effects can be distinguished in Figure 4, which shows the age‐specific increments in height and weight over the period of study. Thus, it contrasts growth in children born 40–60 years apart. In South Korea, the height increment for boys and girls was 5 cm both in infancy and in adulthood, which means that the growth pattern did not change during childhood. This corresponds to a cohort effect, not a period effect. However, the weight increment for South Korean boys was much greater in adulthood than in infancy (13 vs. 2 kg), indicating a strong period effect in addition to the cohort effect. Note that these inferences could have been based either on the observed increments in Figure 4 (solid lines) or the SITAR modeled increments (dashed lines), so the SITAR analysis is useful here but not essential.

As noted at the outset, Europeans have historically been much taller than Asians. The Dutch are recognized as the tallest nation, and their secular increase in height has recently stopped (Schönbeck et al., 2013). Figure 3 of Schönbeck et al. (2013) shows how height in The Netherlands has increased since 1955, equivalent to Figure 4 of the current article. The adult increment amounted to 7 cm in both sexes, but the pattern by age was quite different from Figure 4. The increment increased linearly from birth to 15 years in boys and 11 years in girls, when the increments were respectively 10 and 8 cm, after which they fell back to 7 cm at age 20; so there was no infant increment at all. However, the Dutch in 1955 were already taller (175 and 164 cm by sex) than the South Koreans were in 2005 (Schönbeck et al., 2013), so both the nature of the secular trend and the factors driving it were clearly different in the two countries.

What factors might have caused Japanese and South Korean children to grow so differently from each other? There are several possibilities: genetics (e.g., different height potential), economic status (e.g., per capita gross domestic product or GDP) and diet (e.g., per capita national food supply). Grasgruber, Sebera, Hrazdira, Cacek, and Kalina (2016) explored many factors that might explain the differences in male height in 105 countries. They found that GDP was a strong predictor but national health expenditure was stronger, as was the mortality rate for children under 5 years. This obviously relates directly to living conditions in infancy, when long bone growth is important. Among the strongest predictors was the Human Developmental Index (HDI), which had a correlation of 0.80 with adult height based on 100 countries.

Several dietary factors were predictive, notably animal protein in the form of dairy, eggs, meat and fish, while a rice diet was generally associated with shorter stature (Grasgruber et al., 2016). The strongest single positive influence was dairy protein (*r* = 0.79 for 93 countries), while rice had a strongly negative effect (*r* = −0.74).

So how do Japan and South Korea compare in terms of these predictive factors? GDP was considerably higher in Japan than South Korea, as was the HDI, while child mortality was slightly lower in Japan. (However, the reviewer points out the very different development paths taken by the two countries—Japan achieved a miracle of economic development but then stagnated after 1995, whereas Korea was initially poorer but developed and maintained strong growth, its GDP now matching that for Japan. This may well be relevant.)

In terms of diet, South Koreans ate appreciably less animal protein than Japanese (17 versus 23 g/person/day protein from dairy, eggs, pork and beef). So in all these respects Japan looks healthier than South Korea, and the Japanese ought to be the taller, as predicted by Grasgruber et al. (2016) (their Figure 12). Schwekendiek and Jun (2010) emphasized the impact of primary school feeding programs set up by the South Korean government after the Korean War, which have continued until now. However, Mori compares in detail the national income and dietary intake in the two countries over time, and shows that as recently as 1995, both per capita gross national income and milk intake were three times higher in Japan than in South Korea (Mori, 2017). Mori (2017) also highlights the recent striking increase in South Korean intake of fruit and vegetables, at a time when Japanese intake has fallen, particularly among the young, and this may explain the recent increase in South Korean height.

In conclusion, child height and weight have increased dramatically over the past 40–60 years in Japan and South Korea. The changes have affected both size (i.e., cm and kg) and developmental age, in that the growth period has advanced in timing and shrunk in duration. Most of the height increment seen in adults has already accrued by age 1.5 years, indicating that the secular height trend in these countries (though not in The Netherlands) represents increased growth in the long bones during infancy. The trends have been larger in South Korea than Japan, but there are no convincing differences in national income, diet or lifestyle to explain them.

## CONFLICT OF INTEREST

The authors declare no conflicts of interest.

## Supporting information

Supporting Information Table 1.

Supporting Information Table 2.

Supporting Information Table 3.

Supporting Information Table 4.
