# Supplementary material for: Fifty years of child height and weight in Japan and South Korea: Contrasting secular trend patterns analyzed by SITAR
Source: Am J Hum Biol. 2017 Aug 23;30(1):e23054. doi: 10.1002/ajhb.23054 (PMC5811819; doi:10.1002/ajhb.23054)
Supplement: Supplementary file 1 — Supporting Information Table 1. [file AJHB-30-na-s001.docx]

Supplementary Table 1. Boys height (cm) by age (years) in Japan and South Korea.

JP.1950 JP.1960 JP.1970 JP.1980 JP.1990 JP.2000 JP.2010 KR.1965 KR.1975 KR.1984 KR.1997 KR.2005

1 75.0 77.8 79.8 80.2 80.1 80.6 80.5 74.8 75.8 77.8 77.8 78.9

2 83.4 86.1 88.6 89.1 89.5 89.8 88.9 82.7 85.5 87.9 87.7 90.4

3 90.8 93.2 95.9 96.6 96.8 96.3 97.4 89.0 91.9 94.6 95.7 98.2

4 96.8 99.6 102.4 103.0 104.0 103.4 104.0 95.5 97.9 101.8 103.5 104.7

5 102.8 104.9 108.2 109.4 110.2 109.9 108.7 100.6 105.0 108.4 109.6 111.0

6 108.4 110.6 113.8 115.1 116.4 116.6 116.8 106.7 110.6 113.9 115.8 117.0

7 113.6 116.7 119.8 120.6 122.1 121.5 121.2 112.5 117.7 120.4 122.4 124.9

8 118.2 121.5 124.3 126.1 127.9 128.1 127.9 118.1 122.6 125.6 127.5 130.6

9 122.7 126.4 129.9 131.5 132.2 132.7 132.8 123.7 127.3 130.5 132.9 136.1

10 126.8 130.9 135.0 136.4 137.6 138.4 138.6 128.3 131.9 135.2 137.8 141.3

11 131.1 135.8 139.7 142.8 144.1 144.0 144.4 132.6 136.0 140.3 143.5 147.5

12 135.8 141.1 146.8 149.3 151.5 151.5 151.1 136.7 140.0 144.9 149.3 154.3

13 141.3 147.5 152.8 156.6 158.0 159.2 159.0 143.4 147.5 152.6 155.3 162.0

14 147.0 153.8 159.4 162.9 163.7 164.8 164.4 149.4 153.6 159.2 162.7 167.2

15 152.6 158.5 163.6 166.8 167.2 167.9 169.4 156.2 158.2 164.0 167.8 170.6

16 156.6 161.0 166.1 167.5 168.9 170.1 169.3 162.5 164.1 167.2 171.1 172.2

17 159.0 163.1 167.0 169.6 169.9 170.9 171.1 165.9 166.4 168.3 172.2 173.1

18 160.4 162.8 167.4 169.2 170.9 170.2 171.8 167.8 167.3 168.9 172.5 174.2

19 161.0 163.2 166.9 169.4 170.3 172.2 170.6 168.7 168.1 169.9 173.2 174.5

20 161.5 162.4 166.3 169.9 170.9 170.8 171.1 168.9 168.7 170.2 173.4 174.2
