# Supplementary material for: Fifty years of child height and weight in Japan and South Korea: Contrasting secular trend patterns analyzed by SITAR
Source: Am J Hum Biol. 2017 Aug 23;30(1):e23054. doi: 10.1002/ajhb.23054 (PMC5811819; doi:10.1002/ajhb.23054)
Supplement: Supplementary file 2 — Supporting Information Table 2. [file AJHB-30-na-s002.docx]

Supplementary Table 2. Boys weight (kg) by age (years) in Japan and South Korea.

JP.1950 JP.1960 JP.1970 JP.1980 JP.1990 JP.2000 JP.2010 KR.1965 KR.1975 KR.1984 KR.1997 KR.2005

1 9.4 10.2 10.8 10.9 10.9 11.0 11.1 8.9 9.6 10.3 10.4 10.7

2 11.5 12.1 12.7 12.9 13.0 13.2 12.6 10.8 11.8 12.6 12.9 13.5

3 13.4 14.0 14.5 14.8 15.0 14.8 14.8 12.7 13.3 14.4 15.1 15.6

4 14.9 15.5 16.3 16.5 16.8 16.3 16.7 14.6 14.9 16.0 17.0 17.5

5 16.5 17.0 18.0 18.5 18.9 18.8 17.8 16.0 16.7 18.0 19.0 19.9

6 18.3 18.9 19.9 20.9 21.5 21.9 21.1 16.7 18.5 19.7 21.4 22.4

7 20.2 21.0 22.4 23.0 24.0 24.0 23.7 19.1 20.6 22.3 24.7 26.8

8 22.1 23.1 24.9 26.2 27.0 27.7 26.5 20.4 22.7 24.2 27.6 30.4

9 24.1 25.5 27.5 28.8 29.7 30.9 30.0 23.0 24.9 26.7 31.0 34.5

10 26.0 27.7 30.6 32.0 33.4 34.3 33.8 25.4 27.4 29.5 34.5 38.8

11 28.3 30.5 33.7 36.3 38.5 38.0 37.4 28.8 29.7 32.4 38.6 43.9

12 31.3 34.1 38.7 40.7 43.2 43.5 42.6 31.7 32.2 35.5 42.8 49.5

13 35.1 38.8 43.3 46.3 48.1 49.6 47.3 34.7 37.4 41.5 47.2 55.8

14 39.7 44.2 49.2 52.2 52.9 54.2 52.1 39.7 42.1 47.4 53.9 60.9

15 44.5 49.0 53.2 56.5 57.8 57.2 58.0 44.9 46.1 52.2 58.5 64.9

16 48.4 52.2 56.0 58.1 59.7 60.6 59.1 50.7 52.5 56.2 61.2 66.9

17 51.4 54.7 57.2 61.3 61.7 61.0 61.4 54.5 55.8 58.2 63.2 68.7

18 53.3 55.8 58.2 60.0 62.5 62.0 64.0 57.0 57.1 59.8 63.8 69.6

19 54.4 55.6 57.7 60.4 62.1 62.9 62.8 58.1 58.0 60.2 66.0 70.6

20 55.1 55.5 58.0 61.9 63.6 63.1 63.5 58.2 59.3 61.9 66.6 71.0
