# Supplementary material for: Fifty years of child height and weight in Japan and South Korea: Contrasting secular trend patterns analyzed by SITAR
Source: Am J Hum Biol. 2017 Aug 23;30(1):e23054. doi: 10.1002/ajhb.23054 (PMC5811819; doi:10.1002/ajhb.23054)
Supplement: Supplementary file 3 — Supporting Information Table 3. [file AJHB-30-na-s003.docx]

Supplementary Table 3. Girls height (cm) by age (years) in Japan and South Korea.

JP.1950 JP.1960 JP.1970 JP.1980 JP.1990 JP.2000 JP.2010 KR.1965 KR.1975 KR.1984 KR.1997 KR.2005

1 73.8 76.2 78.9 79.0 78.8 78.8 78.9 72.8 74.8 76.2 76.9 77.6

2 82.2 84.8 87.2 88.2 88.8 87.6 87.2 81.5 84.6 86.9 87.0 89.0

3 89.6 92.0 94.9 95.4 95.6 95.6 94.4 87.7 90.2 92.9 94.2 97.0

4 95.8 98.2 101.8 102.1 103.1 102.5 103.3 94.0 97.1 100.9 102.1 103.4

5 101.7 104.0 107.5 108.5 109.1 109.0 110.3 100.2 103.7 108.1 108.6 109.9

6 107.3 109.8 112.9 114.5 115.2 114.5 115.5 106.5 109.2 113.4 114.7 116.0

7 112.7 115.6 118.4 120.3 121.2 121.1 121.4 112.0 116.9 119.4 121.1 123.7

8 117.5 120.7 124.1 125.3 126.7 127.0 127.2 117.3 121.6 124.9 126.0 129.6

9 121.9 125.8 129.9 131.7 132.7 133.3 134.0 122.0 126.5 130.1 132.2 135.5

10 126.6 131.4 135.4 137.5 138.8 140.1 139.7 128.6 131.8 135.5 137.7 142.3

11 131.6 137.3 142.0 144.4 146.1 145.8 145.2 133.5 137.5 141.8 144.2 148.6

12 136.9 143.3 147.9 149.8 151.1 152.1 152.2 138.7 142.0 147.8 150.9 154.2

13 142.0 147.6 151.2 153.5 154.3 153.7 154.3 144.8 148.1 152.1 155.0 157.5

14 145.8 149.8 153.2 155.1 156.5 156.4 156.0 149.0 152.0 154.9 157.8 159.0

15 148.4 151.5 153.8 156.5 157.4 157.3 157.5 152.9 154.0 155.8 159.0 159.7

16 149.8 151.9 155.1 156.5 157.1 158.5 157.9 154.7 155.6 156.7 160.0 160.4

17 150.5 152.0 154.9 155.9 157.4 157.4 157.7 155.5 156.3 156.6 160.4 160.2

18 150.8 152.4 153.9 156.3 157.8 158.2 157.7 155.7 156.6 157.3 160.5 161.3

19 150.9 152.6 154.4 156.7 158.3 157.7 158.0 155.7 157.0 157.2 160.1 161.6

20 150.8 151.7 154.2 156.6 157.8 157.2 158.1 155.9 157.1 157.6 160.4 161.3
