# Supplementary material for: Fifty years of child height and weight in Japan and South Korea: Contrasting secular trend patterns analyzed by SITAR
Source: Am J Hum Biol. 2017 Aug 23;30(1):e23054. doi: 10.1002/ajhb.23054 (PMC5811819; doi:10.1002/ajhb.23054)
Supplement: Supplementary file 4 — Supporting Information Table 4. [file AJHB-30-na-s004.docx]

Supplementary Table 4. Girls weight (kg) by age (years) in Japan and South Korea.

JP.1950 JP.1960 JP.1970 JP.1980 JP.1990 JP.2000 JP.2010 KR.1965 KR.1975 KR.1984 KR.1997 KR.2005

1 9.0 9.6 10.3 10.5 10.2 10.1 10.1 8.3 9.1 9.5 10.0 10.1

2 10.9 11.6 12.4 12.4 12.5 12.2 12.2 10.3 11.5 12.0 12.5 12.8

3 12.8 13.5 14.1 14.3 14.4 14.3 14.0 12.3 12.8 13.6 14.2 15.1

4 14.4 15.0 15.8 16.3 16.5 16.4 16.3 13.9 14.3 15.7 16.4 16.9

5 16.0 16.5 17.6 18.1 18.4 18.5 18.9 15.5 16.1 17.3 18.4 19.2

6 17.7 18.3 19.5 20.1 20.8 20.5 20.3 17.5 17.9 19.1 20.7 21.5

7 19.5 20.4 21.8 22.8 23.3 23.4 23.0 19.1 20.0 21.2 23.6 25.4

8 21.5 22.6 24.4 24.9 26.5 26.4 26.1 20.9 22.0 23.5 26.2 29.0

9 23.5 25.0 27.3 28.6 29.5 30.6 30.3 23.4 24.2 26.1 30.0 32.7

10 25.8 27.9 30.6 32.3 33.8 35.1 34.3 25.2 27.0 29.2 33.6 37.5

11 28.7 31.7 35.4 36.9 38.9 38.4 37.1 29.1 30.5 33.6 37.8 42.5

12 32.4 36.2 40.4 41.6 42.4 44.9 42.1 32.9 33.6 38.2 43.1 47.3

13 36.6 40.6 44.3 45.1 46.3 46.1 45.6 36.2 38.7 43.1 47.0 50.9

14 40.8 44.3 47.5 48.4 48.7 48.3 47.4 39.8 43.5 46.8 50.7 53.2

15 44.4 47.7 49.6 50.4 50.1 51.0 50.2 44.5 46.7 49.6 52.5 55.2

16 46.8 48.7 50.8 51.7 51.4 52.1 51.9 47.6 49.1 51.2 54.4 55.7

17 48.9 49.7 51.6 51.3 51.6 50.5 51.2 49.6 50.6 51.8 54.6 56.0

18 50.0 50.2 50.9 51.0 51.7 51.5 53.3 50.3 50.8 51.9 54.7 55.4

19 50.5 50.5 51.4 51.2 51.9 50.5 52.6 51.1 51.2 51.5 54.9 55.7

20 50.6 50.3 50.9 50.9 51.1 52.0 52.0 51.5 52.0 51.8 55.7 55.6
